# Sirtuins in glucose and lipid metabolism

**DOI:** 10.18632/oncotarget.12157

**Published:** 2016-09-21

**Authors:** Xin Ye, Meiting Li, Tianyun Hou, Tian Gao, Wei-guo Zhu, Yang Yang

**Affiliations:** ^1^ Department of Biochemistry and Molecular Biology, Peking University Health Science Center, Beijing, China

**Keywords:** sirtuins, SIRT1-SIRT7, glucose metabolism, lipid metabolism, regulation

## Abstract

Sirtuins are evolutionarily conserved protein, serving as nicotinamide adenine dinucleotide-dependent deacetylases or adenosine diphosphate-ribosyltransferases. The mammalian sirtuins family, including SIRT1~7, is involved in many biological processes such as cell survival, proliferation, senescence, stress response, genome stability and metabolism. Evidence accumulated over the past two decades has indicated that sirtuins not only serve as important energy status sensors but also protect cells against metabolic stresses. In this review, we summarize the background of glucose and lipid metabolism concerning sirtuins and discuss the functions of sirtuins in glucose and lipid metabolism. We also seek to highlight the biological roles of certain sirtuins members in cancer metabolism.

## INTRODUCTION

Metabolism plays an important role in every aspect of a cell. When it comes to energy homeostasis, glucose and lipid metabolism are of great importance. Under the condition of calorie restriction, oxidative stress or other energy alterations, glucose metabolism and lipid metabolism are subsequently regulated in response to these alterations [1, 2]. Therefore, molecules that regulate metabolism are highlighted in research for their major roles in cell survival and homeostasis maintenance. Among these regulators, sirtuins are the ones that received significant attention for their important roles in regulating and maintaining glucose and lipid homeostasis [1].

Sirtuins gained their attention as a highly conserved family of NAD^+^-dependent enzymes that extends lifespan in yeast [3]. The mammalian sirtuin family contains seven members (SIRT1-7), which are localized to different cellular compartments and are capable of diverse catalytic activities to modify a great number of proteins, including histone and non-histone proteins [4]. Their biological functions vary from metabolism to cell survival as key regulators and they're involved in a range of diseases, such as diabetes, neurodegeneration and cancer [1]. In this review, we summarize recent progress on the functions of SIRTs and explore the role of sirtuins in glucose and lipid metabolism.

## A BRIEF OVERVIEW OF SIRTUIN FAMILY

The discovery of Sir2, also known as sirtuin, emerged from the studies of how yeast mating type is regulated. Through the studies, four subtypes of sirtuin proteins were defined and their function was identified as a NAD^+^-dependent histone deacetylase to silence the mating type loci [5]. As studies advanced, the sirtuin family is found to be ubiquitous throughout all kingdoms of life, and mammalian sirtuins (SIRT1-7) are ancient in animal evolution with highly conserved structure [5]. However, they are localized in different subcellular compartments: SIRT1, SIRT6, SIRT7 are predominantly in the nucleus, SIRT2 is cytoplasmically localized, and SIRT3, SIRT4, SIRT5 are located in the mitochondria [4].

SIRT1, a NAD^+^-dependent deacetylase of lysine residue of target protein [6], is the most well studied member of the mammalian sirtuin family. Originally it was described as a deacetylase to deacetylate histones [7],but later it was found to have the ability to deacetylate non-histone proteins, such as p53 [8], peroxisome proliferator-activated receptor gamma coactivator 1 alpha(PGC-1α) and forkhead box O1(FoxO1) [9]. SIRT1 plays a significant role in metabolism, influencing mitochondrial biogenesis, glycolysis, hypoxia and angiogenesis [6]. In addition, SIRT1 is associated with genome stability and influences tumorgenesis [7].

SIRT2 is a cytoplasmic protein and it was found to be colocalized with the microtubule network and deacetylate tubulins *in vitro* and *in vivo* [2]. Also, SIRT2 was identified as a deacetylase to deacetylate histone H4 lysine 16 (H4K16) at a global level during mitosis [10]. Dryden et al. found that the abundance of the SIRT2 protein increases during the G2/M transition in Saos2 cells [11]. This accumulation in the G2/M phase has also been observed in U937 cells when over expressing SIRT2 [12]. In addition, the inactivation of SIRT2 may lead to tumorigenesis and thus it is a potential target for cancer therapy [13].

The human SIRT3 sequence includes a functional amino-terminal mitochondrial localization sequence, which enables it to specially locate in mitochondria [14]. As the major mitochondrial deacetylase, SIRT3 targets long-chain acyl CoA dehydrogenase (LCAD), 3-hydroxy-3-methylglutaryl CoA synthase 2 (HMGCS2), isocitrate dehydrogenase 2 (IDH2) and glutamate dehydrogenase (GDH), which function altogether to promote the production of energy in the cell. In addition, SIRT3 can protect the cell from reactive oxygen species (ROS) by activating superoxide dismutase 2 (SOD2) [1]. SIRT4 is an ADP-ribosyltransferase located in mitochondrial matrix. The highly expressed SIRT4 in islet β cells can interact with adenine nucleotide translocator 2/3(ANT2/3) and insulin degrading enzyme (IDE), which down-regulates the secretion of insulin [15]. SIRT5 is a mitochondrial deacetylase. The deacetylation of carbamoyl-phosphate synthase 1(CPS1) by SIRT5 is observed during fasting, which activates ammonia detoxification through the urea cycle [1].

SIRT6 functions as an ADP-ribosylase and NAD^+^-dependent deacylase of both acetyl groups and long-chain fatty acyl groups. It mainly deacetylates histone H3Lys9 [16] and H3Lys56 [17]. Through these functions SIRT6 impacts cellular homeostasis by regulating DNA repair, telomere maintenance and glucose and lipid metabolism [18]. SIRT7 is a widely expressed nuclear protein that is associated with active rRNA genes (rDNA), where it interacts with RNA polymerase I (Pol I) as well as with histones. SIRT7 is also a positive regulator of Pol I transcription and is required for cell viability in mammals [19].

## THE BACKGROUND OF GLUCOSE AND LIPID METABOLISM CONCERNING SIRTUINS REGULATION

The liver is a central metabolic organ that controls several key aspects of lipid and glucose metabolism in response to nutritional and hormonal signals [20]. Insulin is one of the important hormones to regulate glucose metabolism through stimulating the glucose-consuming pathways of glycolysis and glycogenesis. Maintaining lipid homeostasis is a highly complex process which involves lipid storage, synthesis and utilization. Several key regulators are involved in the important process of glucose and lipid metabolism.

### Calorie restriction (CR)

It is established that prolonged calorie restriction may achieve to confer health benefits [21]. By reducing food consumption by 25-60% without malnutrition consistently, CR extends both the mean and the maximum lifespan of rodents, yeast, worms, fruit flies, spiders, birds, and monkeys [22]. It is also found to delay the progression of a variety of age-associated diseases such as cancer, diabetes, cataract, and age-related hearing loss in mammals, and reduces neurodegeneration in animal models of Parkinson's disease and Alzheimer's disease [22]. CR was reported to ameliorate many of the pathologies associated with obesity and metabolic syndrome [22]. Also, CR has been shown to reduce body fat, lower serum triglycerides and LDL cholesterol, raise HDL cholesterol, and increase insulin sensitivity in humans [23].

### Insulin secretion

Pancreatic β cells are of central importance in regulating glucose homeostasis in mammals through secreting insulin in response to elevated blood glucose levels [24]. β cells are able to sense glucose level and transduce its signal to insulin secretion, and there is a highly coordinated mechanism that enables these cells to do so. Glucose enters the β cells via glucose transporter 2(GLUT2) and subsequently enters the glycolysis pathway [25]. Glycolysis increases the ATP/ADP ratio. Subsequently ATP-dependent K^+^ channels are closed, following by the membrane depolarization, resulting in the opening of voltage-gated Ca^2+^ channels. Ca^2+^ flows into the cells and ultimately enables the exocytosis of insulin-containing granules [25]. Mammalian GDH is able to reversibly catalyze amination of L-glutamate to 2-oxoglutarate in mitochondria with the help of NAD^+^ or NADP^+^ as a coenzyme [26]. The allosteric activation of GDH has been reported to result in insulin release through b(-)2-amino-bicyclo [2,2,1] heptane-2-carbocyclic acid (BCH) induction [27]. The stimulation of insulin exocytosis in β cells is associated with the cellular glutamate level. Moreover, high level glutamate activated GDH induces insulin secretion by promoting glutamate oxidation [28]. Also, GDH over-expression in rat islets enables insulin to release at high glucose concentrations. Inhibition of GDH activity decreases insulin release, while activated mutative GDH is associated with a hyperinsulinism syndrome [29].

### Insulin receptors and IGF-1 signaling

There are three insulin/insulin growth factor (IGF) tyrosine kinase receptors: the insulin receptor (IR), IGF-1 receptor, and the orphan IR related receptor. In addition, a structurally and functionally distinct mannose-6-phosphate IGF-2 receptor is thought to have evolved primarily as a scavenger receptor for IGF-2. Insulin, IGF-1 and IGF-2 are the three different ligands for insulin/IGF-1 receptors in mammals. Ligand binding activates IGF-1 or insulin receptor, resulting in the phosphorylation of several intracellular substrates. The phosphorylated substrates then interact with intracellular effectors, including the p85 regulatory subunit of phosphoinositide-3-kinase(PI-3K) and growth-factor-receptor-bound protein-2, thus leading to the activation of the PI3K-PKB/AKT pathway and the Ras-MAPK pathway. These two pathways are highly conserved, and their activation is tightly controlled via a multistep process [30]. The PI3K-PKB/AKT pathway and the Ras-MAPK pathway are two major pathways which respectively regulate the metabolic effects and the mitogenic effects of insulin/IGF-1 signaling [31].

### Lipid synthesis, lipolysis and its regulation

Lipid metabolism is equally important in growth and proliferation. Lipid synthesis involves a variety of lipids essential for cell, including fatty acid, triglycerides, cholesterol, steroids and so on. Lipolysis mainly refers to the process which long-chain fatty acid breaks up into acetyl-CoA, providing energetic needs for cells under physiological circumstances. These two processes are deliberately regulated to achieve a balance that allows cells and tissues to meet their energetic needs as well as the needs for other lipid materials.

One of the most important ways of regulating these processes is by up- or down- regulating the expression of certain transcription factors. These transcription factors target specific genes related to lipid synthesis or lipolysis, and therefore alter the rate of the reaction. One of the most important transcription factors in lipid metabolism is the peroxisome proliferator-activated receptor γ (PPARγ). PPAR is a ligand-activated transcription factor that regulates from development to metabolism [32]. It was first found to exert its effect by binding with retinoid X receptor (RXR) to form heterodimers [33]. PPARγ involves in the transcription regulation of adipocyte differentiation, lipid storage and glucose homeostasis [34]. Hepatic PPARγ is capable of regulating fatty acid uptake, fatty acid trafficking, and triglyceride biosynthesis in the liver by stimulating the expression of related genes controlling these processes [35]. Sterol regulatory element-binding protein (SREBP) is another transcription factor that regulates lipid metabolism. SREBP family includes SREBP-1a, b, c and SREBP-2, sharing 47% of homology [36]. SREBPs directly activate the expression of more than 30 genes dedicated to the synthesis and uptake of cholesterol [37], fatty acids [38], triglycerides, and phospholipids, as well as the NADPH co-factor required to synthesize these molecules [39, 40]. Generally considered, SREBP-1 targets lipogenic genes, such as HMG-CoA, which is a rate-limit enzyme for cholesterol synthesis, as well as fatty acid synthase(FAS) and acetyl-CoA carboxylase (ACC), involving in fatty acid synthesis [41]. SREBP-2 controls cholesterol homeostasis by targeting cholesterol biosynthesis related genes [42]. Through these transcription regulators, lipid metabolism is controlled effectively.

### Metabolic characteristics in cancer cells

Unlimited proliferation is a well-recognized feature of cancer cells, one of the most important demands of which is a continuous energy production. Indeed, certain alterations in glucose and lipid metabolism are observed in cancer cells to adapt to the energy demands. One of the most intriguing phenomenon in cancer cells is its increased rate of aerobic glycolysis, known as the Warburg effect, discovered by Otto Warburg in the 1920s [43]. The Warburg effect describes the enhanced aerobic glycolysis in cancer cells in response to hypoxia conditions or as a result of certain mutations [44]. Changes in oncogenes and tumor suppressor genes take place, resulting in altered metabolic pathways including enhanced glycolysis and repressed oxidative phosphorylation [45]. The swift from oxidative phosphorylation to glycolysis results in more production of lactate, which stimulates angiogenesis and promotes tumor growth [46].

The Warburg effect mainly focuses on the glucose metabolism alterations in cancer cells. In addition, lipid metabolism is also regulated in cancer cells to adjust to the changes. Uncontrolled proliferation also means a great demand for lipids, a main constituent of cellular membranes [45]. In adaption to this requirement, cancer cells increase lipogenesis to the extent that nearly 95% of their saturated and mono-unsaturated fatty acids de novo irrespective of extracellular lipid availability [47]. In physiological conditions, stearoyl-CoA desaturase-1 (SCD1) catalyzed the synthesis of unsaturated lipids, the process of which requires oxygen [48]. Under the condition of hypoxia, however, cells cannot provide themselves with adequate unsaturated lipids. Therefore it leads to endoplasmic reticulum (ER) stress and finally induces the death of normal cells. Hypoxia is a major character of tumor microenvironment, yet cancer cells have evolved to avoid this deadly consequence. Cancer cells generally increase exogenous lipid uptake. Besides, in cancer cells SREBP-1 is up-regulated, resulting in higher expression of SCD1 as well as enhanced lipid uptake [49]. In addition, cancer cells also utilize lipolysis to increase energy production, which will lead to its dysregulation or even dysfunction [50].

## SIRTUINS FUNCTIONS ON GLUCOSE AND LIPID METABOLISM

Except for functioning as transcription factors, sirtuins family is shown to play important roles in the maintenance of glucose and lipid homeostasis, control of insulin secretion and sensitivity, the promotion of fat mobilization, control of oxidative stress, influencing obesity-induced inflammation in macrophages, and the modulation of the activity of circadian clock in metabolic tissues.

### SIRT1

Through its ability to deacetylate target protein, SIRT1 influences cell metabolism by a variety of means, especially in glucose and lipid metabolism (Table 1).

**Table 1:** SIRT1 regulates cell metabolism by interacting with certain factors

Interacting Factor with SIRT1	Effects
CRTC2	glyconeogenesis ↓
FoxO1	glyconeogenesis ↑
PGC-1α	glycolysis ↓; glyconeogenesis ↑; fatty acid use ↑
HIF1α	glycolysis ↓
PGAM-1	glycolysis ↓
SREBP	lipid synthesis ↓; fat storage ↓
AMPK	fatty acid synthesis ↓
PPAR-γ	fatty acid accumulation ↓; fat storage ↓
LXR	cholesterol efflux from cell

### Glucose Metabolism

#### Gluconeogenesis

SIRT1 has a dual but controversial role in the regulation of gluconeogenesis under the condition of calorie restriction. On the one hand, SIRT1 deacetylates CREB-regulated transcription co-activator 2 (CRTC2), leading to CRTC2 degradation and thus decreases hepatic glucose production [51]. On the other hand, SIRT1 activates FoxO1 and PGC-1α to increase hepatic glucose production. SIRT1 activation renders FoxO1 immobile within the nuclear compartment and promotes FoxO1-dependent transcription of genes important for hepatic glucose production [52]. Also, SIRT1 interacts with and deacetylates PGC-1α at specific lysine residues in a NAD^+^-dependent manner and activates gluconeogenic genes and hepatic glucose output through PGC-1α, but does not regulate the effects of PGC-1α on mitochondrial genes [53].

#### Glycolysis

To regulate glycolysis, SIRT1 modulates the effects of PGC-1α repression of glycolytic genes in response to fasting and pyruvate [53]. SIRT1 also suppresses hypoxia inducible factor 1α (HIF-1α) to decrease the rate of glycolysis and to promote oxidative metabolism [1]. In addition, SIRT1 deacetylates phosphoglycerate mutase-1(PGAM-1), reducing its catalytic activity, and therefore inhibits the process of glycolysis [54]. To sum up, under the condition of fasting or calorie restriction, SIRT1 helps to reduce glucose consumption by inhibiting glycolysis.

#### Insulin secretion

SIRT1 is also associated with insulin secretion and sensitivity. Firstly, SIRT1 may function as an insulin sensitizer based on the observed fact that glucose level and hepatic glucose production decreased in SIRT1 transgenic mice as well as in SIRT1 activator-treated mice [55]. Moreover, SIRT1 activation protects from insulin resistance and helps with glucose homeostasis in different insulin sensitive organs [56]. The diabetic patients manifest insulin resistance when the expression and activity of SIRT1 were inhibited in hepatocyte, showing that SIRT1 contributes to hepatic insulin sensitivity [57]. Also, SIRT1 is thought to be a key of regulating skeleton muscle metabolism and that decreased SIRT1 expression may contribute to insulin resistance [54]. In adipose tissue of mice, over-expressed SIRT1 may prevent aging-induced insulin sensitivity reduction [58].

**Figure 1:**
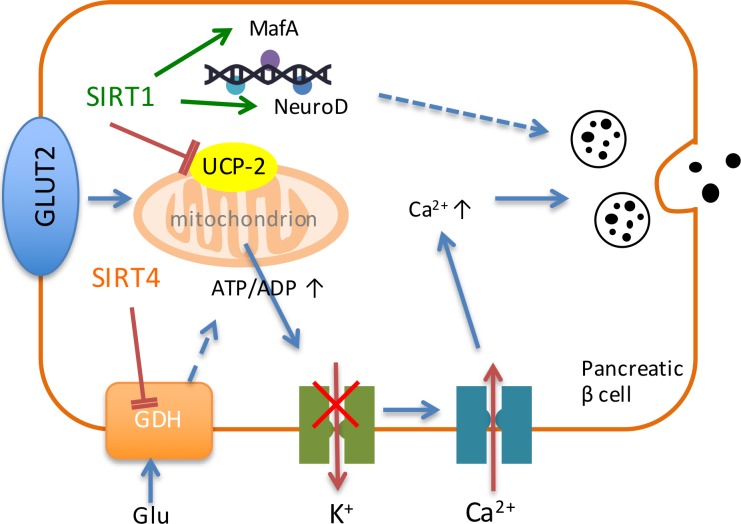
Sirtuins regulate the process of insulin secretion SIRT1 and SIRT4 play vital roles in the regulation of insulin secretion in pancreatic β cells. SIRT1 inhibits the expression of UCP-2 and increases ATP production to shut down the potassium channel, resulting in the influx of calcium and finally the secretion of insulin. Besides, SIRT1 activates the expression of NeuroD and MafA to promote the expression of insulin. SIRT4 down-regulates GDH and generates ATP to promote insulin secretion.

Insulin secretion is upregulated with SIRT1 activation in pancreatic β cells [25]. Pancreatic β cells secrete insulin in response to the elevation of blood glucose level. After glucose enters pancreatic β cells, it breaks down into pyruvate. Then pyruvate enters into mitochondria and produces NADH through tricarboxylic acid cycle, which produces ATP through electron transport chain. SIRT1 inhibits the expression of uncoupling protein 2 (UCP2) and increases the production of ATP. Therefore, the potassium channel is shut and results in the influx of calcium, and finally leads to the secretion of insulin (Figure 1). Under the condition of food deprivation, pancreatic β cells down-regulate the inhibition of UCP-2 by SIRT1 through decreasing the ratio of NAD^+^/NADH and therefore increase the production of ATP and the secretion of insulin [59]. Also, SIRT1 can activate the expression of NeuroD and MafA, two Ins2 gene transcription factors to promote the secretion of insulin (Figure 1) [60].

### Lipid metabolism

SIRT1 regulates a series of proteins and genes related to the process of lipid metabolism. Under the condition of fasting or short-term food deprivation, a metabolic shift from lipid synthesis and storage to lipolysis is observed, the characteristics of which include decreased ATP and NADH level. With further research, SIRT1 was found to repress lipid synthesis and promote lipolysis in response to this typical alteration under the condition of fasting [47]. In the following context presents separately the detailed mechanism concerning how SIRT1 exerts its influence in these two processes in lipid metabolism.

#### Lipid synthesis

It is demonstrated that SIRT1 plays a role in the down-regulation of both SREBP-1 and SREBP-2 during fasting, which results in the inhibition of lipid synthesis and fat storage. SREBPs remain attached to the nuclear envelope and endoplasmic reticulum membranes until they undergo activation. When cellular sterol levels are low, SREBPs undergo cleavage-induced activation and translocate to the nucleus and promote the transcription of enzymes important for sterol biosynthesis [61]. Also, SIRT1 activation by resveratrol correlates with the increase of AMP-activated protein kinase (AMPK), a nutrient sensing molecule, which inhibits fatty acid synthesis [52].

#### Lipolysis

In white adipose tissue, SIRT1 binds to and represses PPARγ activity by docking PPARγ co-repressors, nuclear receptor co-repressor (NCoR) and silencing mediator for retinoid and thyroid hormone receptor (SMRT) [9]. The complex SIRT1/PPARγ/NCoR is recruited to specific DNA sequences in the promoter region of PPARγ target genes and inhibits their transcription. This effect can impact negatively on genes involved in fatty acid accumulation and lipolysis promotion. SIRT1 can alter cellular energy production status by favoring energy mobilization from white adipose tissue and oxidation in tissues like brown adipose tissue [9]. In addition, SIRT1 can be induced in fasting liver and deacetylates PGC-1α to activate fatty acid oxidation genes, which promotes fatty acid use [62].

#### Cholesterol transport

SIRT1 promotes longevity in species ranging from yeast to mammals, and it is believed that these protective actions may result at least in part from the beneficial regulation of energy and metabolic homeostasis [63]. High cholesterol level significantly impacts mortality and a recent study shows an association between cholesterol and SIRT1, which gives another evidence of SIRT1's function to prolong lifespan [64]. SIRT1^−/−^ mice presented significant reduction in total plasma cholesterol level, HDL cholesterol level and triglyceride level, which indicates that SIRT1 is a positive regulator for liver X receptor (LXR). SIRT1 deacetylates LXR on lysine 432 and subsequently promotes its ubiquitination, resulting in the efflux of cholesterol from cells [64]. Activation of LXR is beneficial in that it not only inhibits intestinal cholesterol uptake and promotes reverse cholesterol transport but also exerts potent anti-inflammatory effects that involve in trans-repression. Until now it remains uncertain to what extent the SIRT1-mediated deacetylation of LXR can affect these anti-inflammatory effects [65].

Growing evidence suggests thatSIRT1is a key regulator in glucose and lipid metabolism. It can regulate glucose and lipid metabolismby deacetylating certain proteins through its deacetylase activity. SIRT1might be a new therapeutic target for the prevention of disease related to glucose and lipid metabolism disorder.

### Metabolism in cancer cells

SIRT1 also exerts dual roles in cancer cells. It was initially regarded as a tumor promoter because of its effect on blocking p53-dependent apoptosis [66]. As research advanced, SIRT1 is also found to play a role in tumor suppression by promoting genetic stability and apoptosis through interacting with certain proteins. In addition, SIRT1 is found to promote angiogenesis as well, which is a characteristic in favor of tumor growth [67].

In view of its role in cancer metabolism, SIRT1 tends to be a tumor promoter. It interacts with FoxO1 and activates it through deacetylation [47]. Subsequently, adipose triacylglycerol lipase (ATGL), serving as FoxO1 target gene, is transcriptionally activated [68]. ATGL is a rate-limiting lipolytic enzyme, and its up-regulation will result in increased lipolysis, which meets the energetic demands for the rapidly dividing cancer cells. In other words, SIRT1 indirectly up-regulates ATGL through FoxO1 activation and therefore achieves to increase the rate of lipolysis.

### SIRT2

SIRT2 can mediate phosphoenolpyruvate carboxykinase (PEPCK-C), a rate-limiting enzyme in gluconeogenesis and hence stabilize and decease its ubiquitinylation [69]. Also, SIRT2 is a critical sensor in cellular glucose level. In skeleton muscles, SIRT2 acts as a key component of a signaling network required to maintain the status of insulin resistance and down-regulation of SIRT2 improves insulin sensitivity [70]. More importantly, SIRT2 and its mRNA are highly expressed in fat tissue and it plays an important role in the process of adipocyte differentiation. Under the condition of CR, cold exposure or isoproterenol administration, the expression of SIRT2 is increased in order to regulate adipose function [52]. In addition, SIRT2 mediates adipogenesis by inhibiting PPARγ [52]. FoxO1 can bind to PPARγ or the promoter of PPARγ gene to inhibit PPARγ transcription activity in primary adipocyte [71]. Wang F et al. showed that SIRT2 decreases the acetylation level of FoxO1 and increases its level of binding PPARγ to inhibit adipogenesis through the inhibition of PPARγ [52]. In conclusion, SIRT2 is an important glucose level sensor and maintains insulin sensitivity. Also, it promotes glyconeogenesis or inhibits adipogenesis through its deacetylase activity. SIRT2 plays a role in regulating both glucose and lipid metabolism.

### SIRT3

SIRT3 is the major mitochondrial deacetylase localized primarily in mitochondrial matrix. Several of its targets have been identified, many of which have important roles in metabolic homeostasis [1]. Under the condition of fasting and CR, SIRT3 expression is selectively activated, becoming a metabolic sensor that responds to the changes in the energy status of the cells and modulates the activity of key metabolic enzymes via protein deacetylation [72].

Acetyl-coenzyme A synthetase (AceCS2) is located in mitochondria, which is the first acetylated substrate protein of SIRT3 [73]. Deacetylation of AceCS2 activates the acetyl-CoA synthetase activity of AceCS2 [73]. During fasting, the expression of SIRT3 increases, which leads to the deacetyation of 3-hydroxy-3-methylglutaryl-CoA synthase 2(HMGCS2) to increase in its enzymatic activity [74]. Also, under the fasting condition, LCAD is deacetylated by SIRT3 in liver or in adipose tissues both *in vitro* and *in vivo*, and hyperactylation of LCAD reduces its enzymatic activity. Hence, SIRT3 can deacetylate LCAD to promote fatty acid oxidation [75]. The mitochondrial matrix protein isocitrate dehydrogenase 2 (IDH2) is a key enzyme in tricarboxylic cycle and a major source of NADPH as well. The IDH2 activity can be fully restored to maximum by SIRT3 through deacetylation IDH2 at Lys-413 site [76]. In addition, another substrate of SIRT3 is GDH, which is regulated by reversible ADP-ribosylation. SIRT3 can deacetylate and thereby stimulate GDH enzymatic activity. The N-terminal and C-terminal regions of SIRT3 influence its activity against GDH and peptide substrates, indicating that these regions play important roles in substrate recognition and activity regulation [26]. To sum up, SIRT3 plays a major role in regulating several important metabolism pathways in mitochondria.

### Metabolism in cancer cells

SIRT3 is generally identified as a tumor suppressor by regulating several metabolic pathways. The absence of SIRT3 leads to the overproduction of reactive oxygen species (ROS), which in turn stabilizes HIF-1α in the nuclear and subsequently enhances glycolysis by promoting glycolytic enzymes expression [77]. Moreover, locating in mitochondrial matrix, SIRT3 deacetylates and increases the activity of pyruvate dehydrogenase E1α (PDHA1), resulting in a higher rate of pyruvate transforming into acetyl-CoA. This promotes the utilization of glucose and represses lactate production [78]. These discoveries lead to a conclusion that SIRT3 represses glycolysis and inhibits the production of lactate, which, in other words, is to counteract the Warburg effect. These findings are consistent in various types of cancer. In ovarian cancer cell, SIRT3 down-regulation can promote its metastasis [79]. Moreover, SIRT3 inhibits cancer cell proliferation in prostate cancer and gastric cancer [80, 81]. To conclude, SIRT3 can function as a tumor suppressor by inhibiting the Warburg effect, which indicates its importance in therapeutic approaches.

### SIRT4

**Figure 2:**
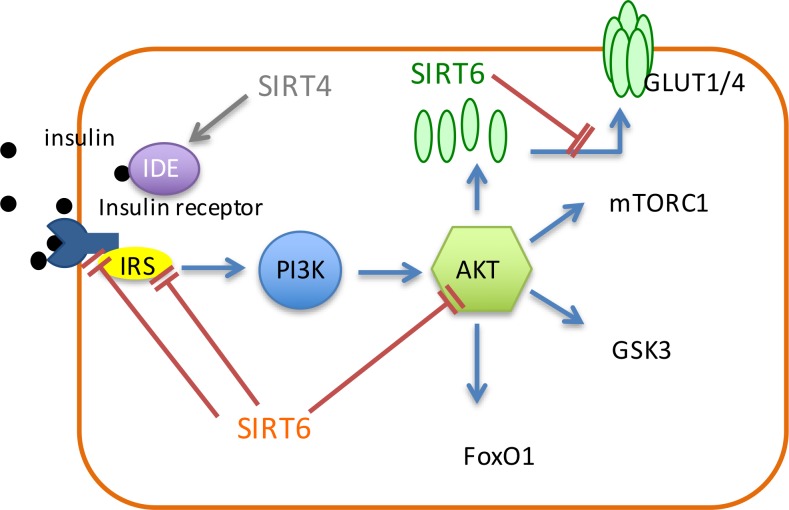
Sirtuins regulate the insulin pathway SIRT6 inhibits insulin receptor, IRS1 and IRS2 to inhibit AKT and insulin signaling. SIRT6 also down-regulates the expression of GULT1/4. SIRT4 interacts with IDE, which is associated with insulin degradation.

SIRT4 was reported to regulate insulin secretion in pancreatic β cells. Under diet conditions, SIRT4 regulates amino-acid-stimulated insulin secretion in β cells by ADP-ribosylating and repressing mitochondrial GDH [82]. Under calorie-sufficient conditions, SIRT4 ADP-ribosylates and down-regulates GDH, which promotes the metabolism of glutamate and glutamine, and generates ATP in β cells to further promote insulin secretion (Figure 1) [82]. During CR condition, there is an increase in GDH activity, which up-regulates insulin secretion in response to glutamine and leucine [82]. Therefore, SIRT4 serves as an insulin secretion repressor under calorie-sufficient conditions. In addition, SIRT4 can interact with IDE (Figure 2) [15], which is associated with type-2 diabetes mellitus [83].

SIRT4 also plays its role in lipid metabolism. The reduced SIRT4 level often results in a significant increase in fatty acid metabolism related enzymes expression [84]. The levels of SIRT4 decrease in the liver and the SIRT4 null mice display an increased expression of PPARα target genes associated with fatty acid catabolism [85].

### SIRT5

Recent studies suggest that SIRT5 regulates protein functions through demalonylation, desuccinylation and glutarylation other than deacetylation [86]. SIRT5 plays a role in regulating intermediary metabolism based on the fact that glycolytic flux is suppressed when SIRT5 is absent in primary hepatocytes, and that SIRT5 regulates the activity of glyceraldehyde-3-phosphate dehydrogenase (GAPDH), a key glycolytic enzyme, via demalonylating the key residue of K184 which located at the enzyme's homodimerization interface [87]. Also, SIRT5 is reported to regulate HMGCS2 desuccinylation at K83 and K310 to restore the binding pocket for phosphate groups of acetyl-CoA to accumulate ketone body production [88].

Although SIRT5 has been found to play multiple roles in regulating cellular metabolism, the identification of its direct substrates and definite functions still needs further research [89].

### SIRT6

SIRT6 is involved in regulating many aspects of cellular metabolism especially in glucose and lipid homeostasis. SIRT6-overexpressing mice fed with a high-fat diet exhibit a decrease in visceral fat accumulation and improvements in blood lipid profile, glucose tolerance, and insulin secretion, and thus SIRT6 exerts its influence in lipid homeostasis [90]. Also, the effect of SIRT6 on cancer suggests a SIRT6-based treatment is viable for at least some specific types of cancer.

### Glucose metabolism

SIRT6 suppresses insulin/IGF-1 like signaling through the inhibition of multiple members in this pathway including AKT, IR, insulin receptor substrate (IRS), glucose transporter-1 (GLUT1) and glucose transporter-4 (GLUT4) (Figure 2). It was demonstrated that SIRT6 negatively regulates AKT phosphorylation through inhibition of IR, IRS1 and IRS2, which consequently leads to the inhibition of insulin signaling and AKT signaling [91]. SIRT6 also maintains glucose homeostasis by down-regulating insulin/IGF-1-like signaling. SIRT6-deficiency causes an increased expression of membrane GLUT1 and GLUT4, and results in hypoglycemia (Figure 2) [92]. Also, SIRT6 deficiency results in the enhancement of H3K9 acetylation and the augment of the promoter binding and the transcriptional activity of c-Jun, which resulted in hyperactivation of multiple IGF signaling [93].

**Figure 3:**
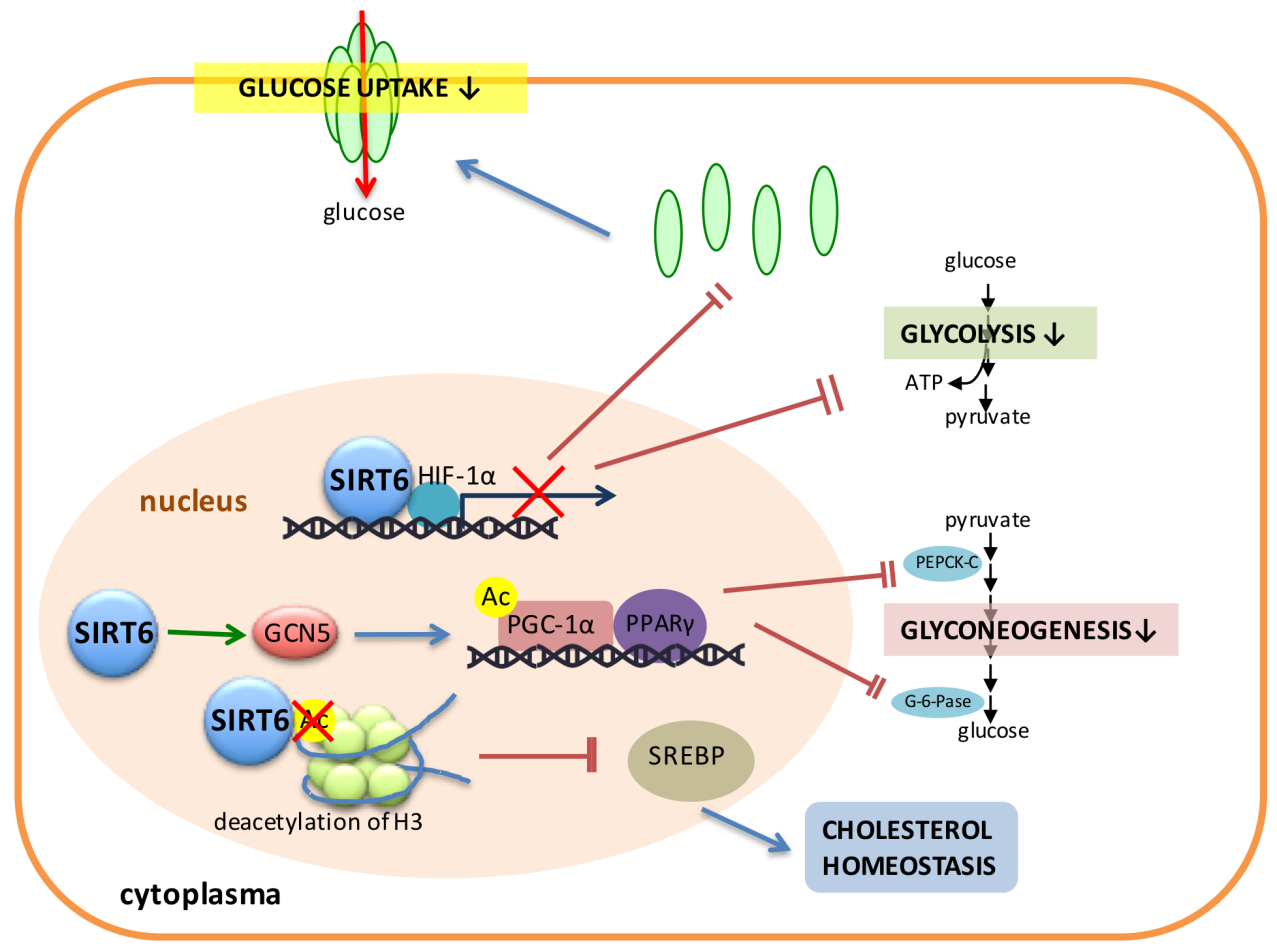
SIRT6 plays a critical role in regulating cell metabolism SIRT6 plays important roles in several pathways concerning glucose and lipid metabolism. SIRT6 binds with HIF-1α and inhibits the expression of glycolytic enzymes and GLUT. SIRT6 also promotes the activity of GCN5, resulting in the acetylation of PGC-1α and the consequent activation of PPARγ to down-regulate the expression of glyconeogenesis-related enzymes such as PEPCK-C and G6P, and subsequently inhibits the process of gluconeogenesis. SIRT6 deacetylates H3 and inhibits the expression of SREBP to regulate the cholesterol homeostasis.

In addition, SIRT6 can down-regulate glycolysis by inhibiting the expression of several key glycolytic genes [94]. Furthermore, SIRT6 appears as a co-repressor of the transcription factors such as HIF-1α, which is a critical regulator of nutrient stress responses (Figure 3). SIRT6 deficient cells exhibit increased HIF-1α activity and increased glucose uptake with up-regulation of glycolysis and diminished mitochondrial respiration [93]. SIRT6 also plays a direct role in controlling glyconeogenesis. For example, SIRT6 interacts with and enhances the activity of General Control Non-repressed Protein 5 (GCN5), which, in turn, catalyzes the acetylation of PGC-1α and the consequent activation of PPARγ to down-regulate the expression of glyconeogenesis-related enzymes such as PEPCK-C and G6P, and subsequently inhibits the process of gluconeogenesis to represse hepatic glucose production (Figure 3) [18].

### Lipid metabolism

Also, SIRT6 deacetylates H3 to further inhibit the expression of SREBP to regulate the cholesterol homeostasis (Figure 3). The cholesterol levels of liver deficient mice were elevated and thus SIRT6 was recruited by FoxO3 to the *Srebp2* gene promoter where SIRT6 deacetylates histone H3 at lysine 9 and lysine56, resulting in the inhibition of the expression of SREBP-2 and its target genes [95].

In addition, SIRT6 binds to and deacetylates H3K9 in the promoter of many genes that are involved in glucose and lipid metabolism, and SIRT6 deficiency results in altered expression of these genes, which ultimately leads to fatty liver in the mutant mice [96]. In other words, SIRT6 absence results in the accumulation of triglycerides while SIRT6 deficiency results in an increased expression of genes responsible for hepatic long-chain fatty acid uptake, a reduced expression of genes for β-oxidation and an increased expression of several genes involved in multiple steps of triglyceride synthesis [96].

### Metabolism in cancer cells

In addition, SIRT6 is identified as a tumor suppressor that regulates cancer metabolism [93]. Alteration in glucose metabolism is now considered as a common feature of cancer and SIRT6 can repress a number of enzymes involved in the homeostatic control of glucose metabolism in cancer cells [93, 97]. As mentioned above, SIRT6 acts as a co-repressor of HIF-1α and subsequently inhibits aerobic glycolysis in cancer cells. Moreover, SIRT6 deacetylates FoxO1, resulting in the inhibition of two rate-limiting enzymes for glyconeogenesis glucose-6-phosphase (G6P) and PEPCK-C. The process can be enhanced by p53 in cancer cells through promoting the expression of SIRT6, and therefore inhibits glyconeogenesis and represses tumor cell growth [98]. On the contrary, SIRT6 suppression was observed in several types of cancer cells and therefore the proliferation of the tumor cells is accelerated [99, 100]. Low expression of SIRT6 in bladder cancer cells results in enhanced glucose uptake and lactate production by promoting the expression of GLUT1 and pyruvate dehydrogenase kinase (PDK1), two key glycolysis proteins [100]. With further research, E2F1 represses SIRT6 and subsequently increases GLUT1 and PDK1 expression, resulting in glycolysis enhancement [101]. Following the same mechanism, SIRT6 in breast cancer cells is repressed by runt-related transcription factor 2 (RUNX2) transcription factor, and thus results in a higher rate of glycolysis and the inhibition of mitochondrial respiration [99]. To sum up, SIRT6 is repressed by certain molecules in various types of cancer cells, resulting in a higher rate of glycolysis and hence promotes tumor progress [99, 100]. Yet, the role of SIRT6 as a tumor suppressor is challenged by the fact that SIRT6 promotes COX-2 expression and acts as an oncogene in skin cancer, suggesting a greater complexity to its role in epithelial carcinogenesis [102].

### SIRT7

Several recent studies have shed new light on the function of SIRT7 associated with energy metabolism. Specifically, three new studies show that SIRT7 exerts a vital role in hepatic lipid metabolism, yet their conclusions seem contradictory. Shin et al reported that SIRT7 KO mice presented with liver steatosis, elevated triglyceride level and increased expression of lipogenic genes [103]. The underlying mechanism for their observations is that c-Myc recruits SIRT7 and suppresses ER stress. SIRT7 deficiency hepatocytes have increased ER stress and therefore developed liver steatosis, resulting in the subsequent dyslipidemia [103]. Ryu et al observed the likewise results and came up with a different mechanism that SIRT7 deacetylates GA-binding protein 1(GABP1), which is a regulator of multiple nuclear-encoded mitochondrial genes, and therefore affects the mitochondrial homeostasis [104]. Yoshizawa et al presented the contradictory findings that SIRT7 KO mice exhibited resistance to high fat diet-induced liver steatosis, obesity and glucose intolerance, and showed significantly lower hepatic triglyceride content [105]. They found that SIRT7 can interact with an DDB1-CUL4-associated factor 1 (DCAF1)/damage-specific DNA binding protein 1 (DDB1)/cullin 4B (CUL4B) E3 uniquitin ligase complex, then inhibits testicular receptor 4 (TR4) degradation and thus promotes fatty acid uptake and triglyceride biosynthesis and storage [105]. Also, SIRT7 is reported to interact with HIF-1α and HIF-2α, two regulators of energy metabolism, resulting in the inhibition of their transcriptional activity and a decrease in their protein levels [106].

In addition, SIRT7 exerts its influence on cancer metabolism. A study of drug-resistant cancer cells showed that SIRT7 inhibition increases stress resistance and modulates insulin/IGF-1 signaling pathways [107].

## CONCLUSIONS

Each member of sirtuin family has exerts its own role in regulating glucose and lipid metabolism under different physiological circumstances. In this review, we summarize the evidence to date and try to present a general overview of sirtuins in metabolism regulation from our current knowledge.

Sirtuins protein initiates the influence in metabolism by sensing metabolic changes resulting from physiological changes in cells. These changes will promote sirtuins interacting with certain transcriptional factors or cofactors, resulting in changes in transcriptional level of target genes associated with metabolism. Sirtuins can also interact with enzymes that are directly involved in metabolism reactions and alter the enzymatic activity, achieving the metabolism regulation. Most sirtuins have wide distribution in various tissues and organs, including liver, brain, skeleton muscle, pancreas, skeleton and so on. However, certain members of sirtuins locating in different tissue or organ can exhibit different effect on metabolism due to the difference in transcription factors expressed in different cells. SIRT1/3 plays important roles in CR. Under the condition of CR, SIRT1 exerts different influences in different locations. It represses PPARγ, inhibits fatty acid accumulation and promotes lipolysis in the cellular matrix, while in mitochondria it promotes FoxO1 and PGC-1α to accelerate hepatic glucose production. SIRT3 deacetylates and increases the enzymatic activity of IDH2 and LCAD to promote fatty acid oxidation and tricarboxylic cycle. SIRT1/3 also plays the key roles in CR-related diseases such as age-associated disease, Parkinson's disease and Alzheimer's disease. Moreover, SIRT3/4/5 exists in mitochondria as deacetylase that function as metabolism regulators, which suggests their relevance of the treatment of inherited diseases related to mitochondrial metabolism. In addition, several sirtuins including SIRT6 and SIRT7 are found to be critical regulators for cancer cells metabolism and therefore further studies should be implemented to indicate whether the modulation of sirtuins have implications for cancer treatment. The increasing knowledge about sirtuins shows that sirtuins play important roles in regulating the glucose and lipid homeostasis. To further determine the metabolic functions of sirtuins will make it easy to understand the metabolic diseases and it will be beneficial for development of novel therapies that target members of sirtuin family.

## Terminology and abbreviations

ACC: acetyl-CoA carboxylase
ACL: ATP citrate lyase
AceCS2: acetyl-coenzyme A synthetase
AKT: protein kinase B
AMPK: AMP-activated protein kinase
ANT: adenine nucleotide translocator
ATGL: adipose triacylglycerol lipase
BCH: b(-)-2-amino-bicyclo [2,2,1]-heptane-2-carbocyclic acid
CPS1: carbamoyl phosphate synthetase 1
CR: calorie restriction
CRTC2: CREB-regulated transcription co-activator 2
CUL4B: cullin 4B
DCAF1: DDB1-CUL4-associated factor 1
DDB1: damage-specific DNA binding protein
ER: endoplamic reticulum
FAS: fatty acid synthase
FATP: fatty acid transport protein
FGF21: fibroblast growth factor 21
FoxO1: forkhead box O1
FXR: farnesoid X receptor
G6P: glucose-6-phosphase
GCN5: General Control Non-repressed Protein 5
GDH: glutamate dehydrogenase
GLUT: glucose transporter
GR: glucocorticoid receptor
GRE: GR respond element
HIF-1α: hypoxia-inducible transcription factor 1α
HMG-CoA: 3-hydroxy-3-methylglutaryl-coenzyme 1
HMGCS2: 3-hydroxy-3-methylglutaryl CoA synthase 2
IDE: insulin degrading enzyme
IDH2: isocitrate dehydrogenase 2
IGF: insulin-like growth factor
IR: insulin receptor
IRS: insulin receptor species
LCAD: long-chain acyl CoA dehydrogenase
LPL: lipoprotein lipase
LXR: liver X receptor
MAPK: mitogen-activated protein kinase
NCoR: nuclear receptor corepressor
PDK: pyruvate dehydrogenase kinase
PEPCK-C: phosphoenolpyruvate carboxykinase
PGAM-1: phosphoglycerate mutase-1
PGC-1α: peroxisome proliferator-activated receptor γ coactivator 1α
PI-3K: phosphoinositide-3-kinase
PPARγ: peroxisome proliferator-activated receptor γ
RCT: reverse cholesterol transport
ROS: reactive oxygen species
RXR: retinoid X receptor
SCD1: stearoyl-CoA desaturase-1
SHP: small heterodimer partner
SIRT: sirtuin
SMRT: silencing mediator for retinoid and thyroid hormone receptor
SOD2: superoxide dismutase 2
SREBP: sterol regulatory element-binding protein
TCA: tricarboxylic acid
UCP2: uncoupling protein 2.
